# Conditioned media of deer antler stem cells accelerate regeneration of alveolar bone defects in rats

**DOI:** 10.1111/cpr.13454

**Published:** 2023-03-16

**Authors:** Qianqian Guo, Junjun Zheng, Hongbing Lin, Zhongming Han, Zhen Wang, Jing Ren, Jingjie Zhai, Haiping Zhao, Rui Du, Chunyi Li

**Affiliations:** ^1^ Institute of Antler Science and Product Technology, Changchun Sci‐Tech University Changchun Jilin China; ^2^ Institute of Special Economic Animal and Plant Sciences, Chinese Academy of Agricultural Sciences Changchun Jilin China; ^3^ Jilin Provincial Key Laboratory of Tooth Development and Bone Remodeling, Hospital of Stomatology Jilin University Changchun Jilin China; ^4^ Jilin Agricultural University, College of Chinese Medicinal Materials Changchun Jilin 130118 China; ^5^ Department of Oral Implantology Jilin Provincial Key Laboratory of Sciences and Technology for Stomatology Nanoengineering, Hospital of Stomatology, Jilin University Changchun Jilin China; ^6^ Qingdao Agricultural University, College of Animal Science and Technology Qingdao Shandong China

## Abstract

The destruction of periodontal alveolar bone (AB) caused by periodontitis is regarded as one of the major reasons for tooth loss. The inhibition of bone resorption and regeneration of lost AB are the desirable outcomes in clinical practice but remain in challenge. The use of mesenchymal stem cells (MSCs) is one current approach for achieving true restoration of AB defects (ABD). Antler stem cells (AnSC) are capable of renewing a huge mammalian bony appendage, the deer antler, suggesting an unparalleled potential for bone regeneration. Herein, we investigated the effectiveness of deer AnSCs conditioned medium (CM, AnSC‐CM) for repair of surgically‐created ABD using a rat model and sought to define the underlying mechanisms. The results showed that AnSC‐CM effectively induced regeneration of AB tissue; the outcome was significantly better than human bone marrow mesenchymal stem cell conditioned medium (hBMSC‐CM). AnSC‐CM treatment upregulated osteogenic factors and downregulated osteoclastic differentiation factors; stimulated proliferation, migration and differentiation of resident MSCs toward osteogenic lineage cells; modulated macrophage polarization toward the M2 phenotype and suppressed osteoclastogenesis. That AnSC‐CM resulted in better outcomes than hBMSC‐CM in treating ABD was attributed to the cell compatibility as both AnSCs and AB tissue are neural crest‐derived. In conclusion, the effects of AnSC‐CM on AB tissue regeneration were achieved through both promotion of osteogenesis and inhibition of osteoclastogenesis. We believe that AnSC‐CM is a candidate for effective treatment of ABD in dental clinical practice but will require investment in further development.

## Graphical abstract

Schematic illustration of the molecular mechanism underlying the effective treatment of the ABD by AnSC‐CM. Surgical creation of the ABD promoted the homing of endogenous MSCs, PDLSCs and immune cells to the injured area, and induced polarization of circulating monocytes toward M1 type (pro‐inflammatory) and osteoclastogenesis, resulted in bone resorption. Application of AnSC‐CM on the defects resulted in more AB tissue regeneration than the control. AnSC‐CM treatment to the ABD area, on one hand, induced homing MSCs to different into osteoblasts and elevated expression levels of osteogenic genes for the promotion of new bone tissue formation; on the other hand, induced circulating monocytes to polarize to anti‐inflammatory type M2 macrophage and stimulated expressions of anti‐inflammatory genes to further enhance osteogenesis via osteoblast differentiation; and at the same time, inhibition of polarization of pro‐inflammatory M1 macrophage and expressions of pro‐inflammation factors effectively suppressed osteoclastogenesis via inactivation of NFκB signalling pathway. Overall, the effects of AnSC‐CM on AB tissue regeneration were achieved through both stimulation of osteogenesis and inhibition of osteoclastogenesis.

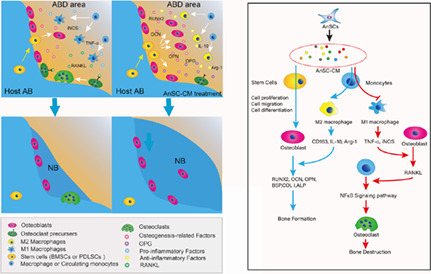

## 1 INTRODUCTION

Destruction of periodontal tissues, including gingiva, root cementum, periodontal ligament (PDL) and alveolar bone (AB), is caused by periodontitis, an acute or chronic oral inflammatory disease.^1^ Periodontitis can destroy tooth‐investing tissues and lead to tooth loss if left untreated.^2^ An epidemiological survey showed that periodontitis has high prevalence worldwide and more than half of all adults are affected by this disease to varying degrees.^3^, ^4^ This problem is expected to deteriorate further as a result of on‐going demographic change.^5^ Despite its widespread prevalence in the general population, traditional therapies have achieved only limited success in treating intrabony periodontal damage, such as ABD.^6^

The use of mesenchymal stem cells (MSCs) has become one of the viable approaches for effective periodontal tissue repair, particularly for complete regeneration of the periodontal tissue complex.^7^, ^8^ MSCs are known to be able to self‐renew and differentiate into multiple cell types and thus have tremendous therapeutic potential. For example, MSCs locally injected into mouse ABD effectively regenerated new bone tissue.^9^, ^10^ Among various MSCs used for periodontal tissue regeneration, PDL stem cells (PDLSCs) have proven to be the most effective type for this purpose in vivo.^11^, ^12^, ^13^ However, there are some problems associated with the use of PDLSCs, such as a limited source, variation in stem cells due to donor quality and the painful process of collection.^14^, ^15^

It is known that periodontal tissues originate from cranial neural crest‐derived ectomesenchyme during embryo development,^16^, ^17^, ^18^ whereas most other types of MSCs used for treating intrabony defects of periodontium are mesoderm‐derived, such as bone marrow‐MSCs (BMSCs) and adipose‐MSCs (AMSCs).^7^ According to Leucht et al., MSCs have ‘positional memory’, which influences how the cells behave when grafted into ectopic locations.^19^ When the neural crest‐derived bone is injured, the healing callus is composed entirely of neural crest‐derived cells, whereas when the tibia is damaged, the injury site is occupied entirely by mesoderm‐derived cells. Indeed, when craniofacial defects are repaired using mesoderm‐derived cells (e.g., the fibula, iliac crest, ribs), the outcome is less effective than grafts of neural crest‐derived cells.^20^ If this is the case, other types of neural crest‐derived MSCs may be more effective than BMSCs or AMSCs in repairing periodontal damage.

Deer antlers are the only mammalian appendage that, once lost, can fully regenerate.^21^, ^22^, ^23^ Antler regeneration relies on the periosteum of the pedicle (PP), a bony protuberance permanently covered by skin from which antlers are cast and regenerate annually.^24^, ^25^ Each year, around 3.3 million PP cells give rise to up to 15 kg of antler tissue mass in around 3 months.^26^ As PP cells have stem cell attributes, they have been termed antler stem cells (AnSCs).^27^, ^28^ Interestingly, AnSCs are also neural crest‐derived.^29^, ^30^ This means it may be possible to harness the proliferation and differentiation potential of AnSCs to induce regeneration of damaged periodontal hard tissue, that is, AB.

Although transplanted MSCs are expected to generate new tissues through their engraftment, proliferation and differentiation, it has been shown that transplanted MSCs have a relatively short life and limited engraftment in the recipient sites,^31^ which also clearly manifests in cases of periodontal tissue repair.^14^, ^32^ Recent studies have shown that it is the paracrine effects of implanted MSCs that promote tissue regeneration in vivo.^33^, ^34^ Thus, conditioned medium, containing most of the paracrine factors from cultured MSCs (MSC‐CM), can induce tissue regeneration as effectively as MSCs.^35^ Besides, there are advantages to using MSC‐CM over the MSCs themselves including a lower risk of tumorigenesis, easier preservation and handling and negligible immunogenicity which lowers the hurdles associated with allo‐ or xeno‐transplantation.^14^

The aims of the present study were (1) to determine the effectiveness of deer AnSC conditioned medium (AnSC‐CM) for repairing surgically created ABD in comparison to BMSCs using a rat model; (2) to reveal the molecular mechanism underlying the effective treatment of AnSC‐CM on rat ABD through an in vitro approach; (3) to assess whether the effects of AnSC‐CM on rat BMSCs also apply to human PDLSCs and (4) to assess the similarities and contrasts in effects between neural crest‐derived and mesoderm‐derived MSCs in regenerating AB tissue. Positive results would help pave the way for devising a novel cell‐free therapeutic for repairing ABD in the clinical setting.

## 2 MATERIALS AND METHODS

### 2.1 Cell culture

PP tissue was obtained in a previous study.^36^ Procedures for culturing the PP cells, that is, antler stem cells (AnSCs), were conducted as described previously.^28^ Human bone marrow mesenchymal stem cells (hBMSCs) were purchased from Guangzhou Cellbank Biotech Co., Ltd (Guangzhou, China). Human periodontal ligament stem cells (hPDLSCs) were gifted by Dr. Yuqin Shen from Guangzhou Medical University. Rat bone marrow mesenchymal stem cells (rBMSCs) and rat bone marrow monocytes (rBMMs) were isolated from SD rats as previously described.^37^, ^38^ Briefly, the femurs and tibias were obtained aseptically, after cutting off both ends of the bones, marrow cells were flushed out using DMEM medium. To remove red blood cells, the marrow cells were treated with red blood cell lysis buffer (Solarbio, Beijing, China) following the manufacturer's instructions. After washing, the cells were cultured in 60‐mm cell culture dishes containing complete medium (DMEM [Life, USA] plus 10% FBS [Gibco, USA], 100 U/mL penicillin and 100 μg/mL streptomycin [Invitrogen, USA]) at 37°C in a 5% CO_2_ incubator. The suspended cells, that is, rBMMs, were collected on the second day and reseeded in complete medium supplemented with 20 ng/mL M‐CSF. The adherent cells, that is, rBMSCs were continuously cultured in the complete medium. After a 3‐day culture, non‐adherent lymphocytes were removed via medium change. Cell morphologies of rBMSCs and rBMMs were examined under a microscope.

All types of cells were cultured in the complete medium at 37°C in a 5% CO_2_ incubator, passaged using trypsin (Sigma, USA) and stored in liquid nitrogen in the freezing medium (90% FBS + 10% DMSO). When required, cells were thawed and seeded in T75 flasks (Nest Biotechnology, USA).

### 2.2 Preparation of conditioned mediums

The production of the conditioned medium (CM) was performed using the 5th passage AnSCs and hBMSCs as previously described.^39^ Briefly, when AnSCs and hBMSCs reached 90% confluence, the complete medium was discarded and the cells were washed twice with phosphate‐buffered saline (PBS; Gibco, USA). After incubation in the α‐MEM medium (without FBS and P/S) for 48 h, the supernatants were harvested and centrifuged at 1000 rpm at 4°C for 10 min and concentrated using ultrafiltration with a cut‐off molecular weight at 3 kDa (5000 g, 40 min at 4°C; Millipore Corp, Billerica, MA). The resultant CMs were filtered through 0.22‐μm pore size filters (Millipore Corp, Billerica, MA) and further concentrated via lyophilization, and finally rehydrated. The total protein concentration of each type of CM was measured using the BCA Protein Assay Kit (Beyotime, China): AnSCs‐CM 60 μg/μL; hBMSCs, 54 μg/μL, and finally adjusted to 50 μg/μL as the stocking solution and stored at −80°C for the subsequent experiments. The control medium (α‐MEM) was concentrated and rehydrated following the same procedure as above.

### 2.3 Animal experiments

The design of the animal experiments was approved by the Institutional Animal Care and Use Committee of Changchun Sci‐Tech University (No. CKARI202109), and all experimental animals were treated accordingly. For creation of the ABD, 64 SD rats were randomly divided into four groups: (1) Control: defects with treatment of 10 μL PBS soaked in the collagen membrane (colM; Bio‐Gide®, Geistlich, Switzerland) that had been trimmed to a pre‐determined size of 3 mm × 2 mm; (2) α‐MEM: defects with treatment of 10 μL α‐MEM soaked in the colM; (3) AnSC‐CM: defects with the treatment of 10 μL AnSC‐CM (500 μg/rat) soaked in the colM and (4) hBMSC‐CM: defects with treatment of 10 μL hBMSC‐CM (500 μg/rat) soaked in the colM. On day 3, 7 and 10 after surgery, each group of animals was locally injected with 10 μL PBS, α‐MEM, hBMSC‐CM and AnSC‐CM, respectively.

**FIGURE 1:**
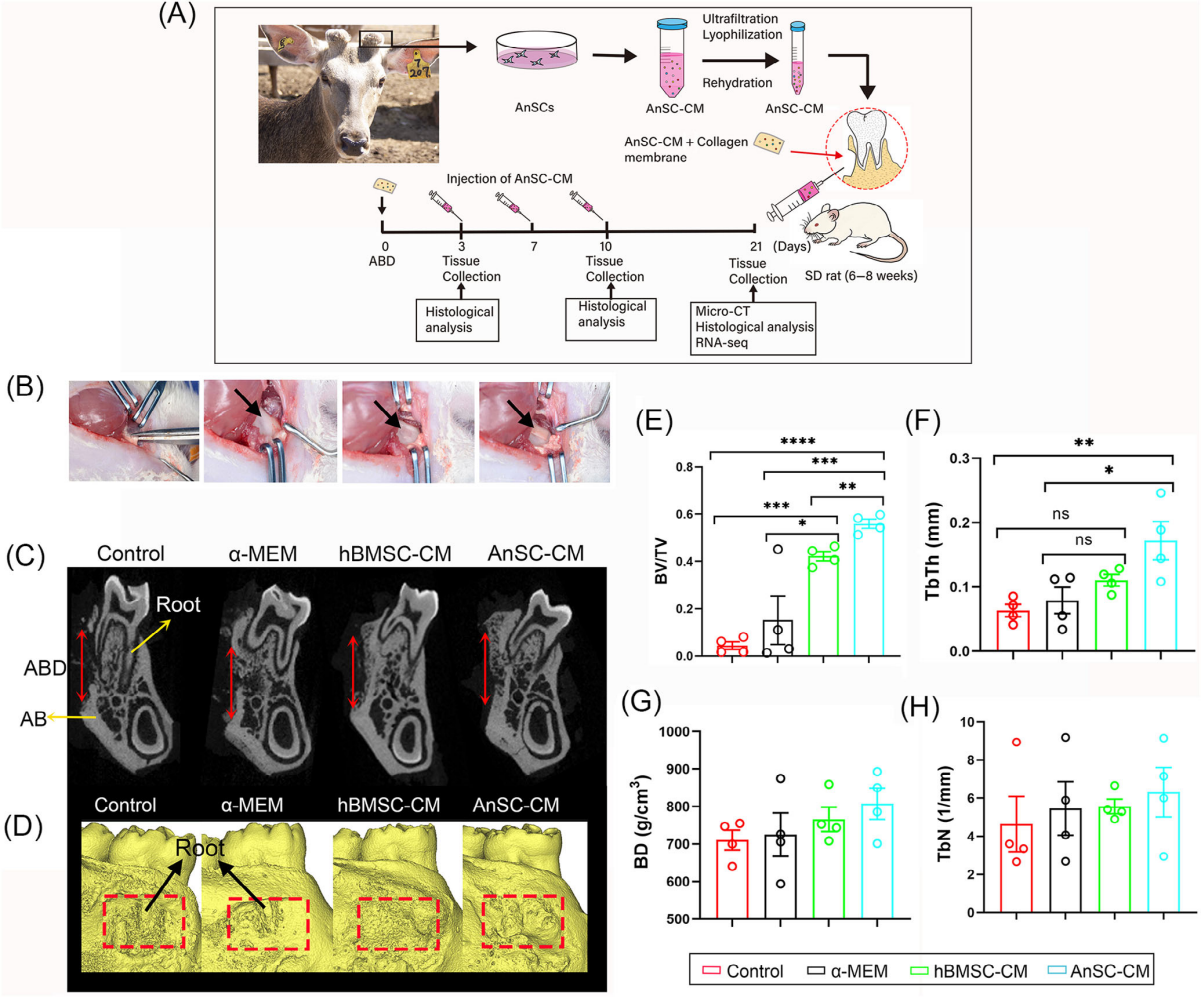
Experimental procedure, surgical creation of alveolar bone defect (ABD), and effects of AnSC‐CM on ABD repair in vivo in rats. (A) Schematic of production of AnSC‐conditioned medium (AnSC‐CM) and application of AnSC‐CM on the ABD. (B) Surgical creation of ABD (arrows) and topical application of AnSC‐CM‐soaked collagen membrane (colM; arrow). (C,D) Reconstructed images of micro‐CT. Note that AnSC‐CM treatment had visibly enhanced regeneration of AB, in comparison with the other groups; and tooth roots had been covered by the newly regenerated new bone (NB) in the hBMSC‐CM and AnSC‐CM groups (D). Red arrows in (C) denote the length of ABD. (E–H) Comparisons of parameters of the NB in different groups: bone volume/tissue volume (E; BV/TV), average thickness of bone trabecula (F; TbTh), bone density (G; BD), and average number of bone trabeculae (H; TbN). Note that treatments with both hBMSC‐CM and AnSC‐CM had significantly increased BV/TV; notably, AnSC‐CM treatment was more significantly increased value of BV/TV than the hBMSC‐CM treatment. Data is shown as mean ± SEMs; *n* = 4, *p < 0.05; **p < 0.01; ***p < 0.001; ****p < 0.0001.

The surgery was carried out as described previously.^13^ Briefly, general anaesthesia was induced via intraperitoneal injection of 60 mg/kg sodium pentobarbital (Sigma‐Aldrich, USA). The incision was made on the skin of the right orofacial side in an anterior–posterior direction to gain access to the mandible. The defect on the AB was made using a low‐speed dental drill with profuse irrigation of saline till the roots of first molars were exposed. The dimension of the defect was ~3 mm × 2 mm × 1 mm. The wound was carefully irrigated and treated. Finally, the skin incision was sutured. All animals were injected with penicillin daily for 3 days after surgery. On day 3 and 10 after surgery, 3 animals were sacrificed from each group and relevant tissue samples were collected for subsequent histological analysis. On day 21, 10 animals were sacrificed from each group and tissue samples were collected for histological analysis, RNA‐seq and micro‐CT analysis (Figure 1A).

### 2.4 Micro‐CT scanning

Right mandibles were collected from each of four animals killed on day 21 after surgery and were scanned using a high‐resolution Micro‐CT (Scano Medical AG, Bassersdorf, Switzerland) using an acquisition protocol (70 kV, 150 mA and 10‐μm increments). The scanned files were reconstructed and analysed using Mimics Innovation Suite software (Materialize, Belgium). The tissue volume selected for quantification analysis was fixed in the shape and size and was in the same area for each sample to minimize error.

### 2.5 Histology and immunohistochemistry

Rat tissue samples were fixed in 4% paraformaldehyde (PFA) and decalcified in 5% methanoic acid for 1 week. Deer PP tissue and rat tissue samples were embedded in paraffin and cut at 5‐μm thickness. For histology, the sections were deparaffinized, rehydrated, and stained with haematoxylin–eosin (H&E), Masson's trichrome and immunohistochemistry (IHC). Masson's trichrome staining was performed using commercial kits (Solarbio, Beijing, China) according to the manufacturer's protocols. In immunohistochemistry, primary antibodies included rabbit anti‐rat RUNX2 (1:500, Bioss, bs‐1134R), rabbit anti‐rat BMP4 (1:200, Abcam, ab118867), rabbit anti‐rat NFATc1 (1:500, Affinity, DF6446), rabbit anti‐rat CD163 (1:500, abcam, ab182422), rabbit anti‐rat iNOS (1:500, Abcam, ab283655), rabbit anti‐rat OPG (1:500, Bioss, bs‐0431R) and rabbit anti‐rat RANKL (1:500, Bioss, bs‐0747R). Images were captured under an inverted microscope (Nikon, Japan) or slice scanner (M8 microscope and scanner, PreciPoint, Germany). The number of RUNX2^+^, NFATc1^+^ and CD163^+^ cells per section were counted manually. For the staining of iNOS, OPG and RANKL, the mean optical density (MOD) values were defined as IOD/area (the area indicated the total number of pixels in the picture) using Image‐Pro Plus software.

### 2.6 Cell proliferation and colony formation assays

Cell proliferation was measured using the Cell Counting Kit‐8 (Solarbio, Beijing, China) according to the manufacturer's instructions. The colony formation assay was carried out using a reported methodology.^36^ Briefly, 200 cells in 2 mL medium were seeded into each well of 6‐well plates and medium was replaced every 4 days after seeding; 14 days after culture the formed cell colonies were washed with PBS, fixed in 4% PFA for 30 min, and stained with 0.5% crystal violet dye for 5 min. Non‐specific staining was removed via three rinses with double‐distilled water, and the cells were photographed under a microscope (EVOS M5000, Thermo Fisher, USA).

### 2.7 5‐Ethynyl‐20‐deoxyuridine incorporation assay

5‐Ethynyl‐20‐deoxyuridine (EDU) incorporation assay was performed using a keyFluor594 Click‐iT EDU Kit (KeyGEN BioTECH, China) according to the manufacturer's protocol. Briefly, cells were first incubated in the medium supplemented with 50 μM EDU for 3 h and fixed in 4% PFA for 30 min. The cells were then permeabilized with 0.5% Triton X‐100 for 10 min and subsequently blocked with 3% bovine serum albumin (BSA) in PBS for 2 h. The nuclei of cells were counterstained with DAPI for 5 min in the dark. The specific fluorescent staining was examined under a fluorescent microscope (EVOS M5000, Thermo Fisher, USA).

### 2.8 Cell migration assay

rBMSCs were seeded in 24‐well plates at a density of 2 × 10^4^ cells/well and incubated in the complete medium to allow cell adhesion and formation of a confluent monolayer. These confluent monolayers were then scratched with a sterile pipette tip to leave a space ~0.4–0.5 mm in width. Culture medium was then immediately removed (along with any dislodged cells). The medium was replaced by (1) a fresh serum‐free medium supplemented with α‐MEM, or (2) a fresh serum‐free medium supplemented with AnSC‐CM (0.2 μg/μL; 100 μg/500 μL/well), or (3) a fresh serum‐free medium supplemented with hBMSC‐CM (0.2 μg/μL; 100 μg/500 μL/well). Images were captured using an inverted microscope camera at various times after the scratch. The digitized images were then analysed using Image‐J software to measure the width of the space. Cell migration rate was calculated as the percentage by which the original scratch width decreased at each given time point.

### 2.9 Osteogenic induction and Alizarin red staining

BMSCs and PDLSCs were seeded at a density of 5 × 10^5^ cells/well in 6‐well culture plates. When the cells reached 80% confluence, the media were changed to osteogenic induction media (α‐MEM, AnSC‐CM, hBMSC‐CM supplemented with 50 μg/mL vitamin C, 10 nM dexamethasone and 10 mM ß‐glycerophosphate, all purchased from Solarbio, China); medium was refreshed every other day. On 14 and 21 days of osteogenic induction, cells were fixed in 4% PFA for 15 min and stained with 0.2% Alizarin Red S (Solarbio, Beijing, China) for 30 min at room temperature. After washing twice with double distilled water, surface staining was photographed under a microscope. For quantification, the mineralized nodules were dissolved in 500 μL 2% cetylpyridinium chloride for 1 h at room temperature, and the OD values of the solutions were measured at 562 nm.

### 2.10 ALP staining

BMSCs and PDLSCs were seeded at a density of 2 × 10^4^ cells/well in 24‐well culture plates, and osteogenic induction performed as described in the section ‘Osteogenic induction and Alizarin red staining’. After 7 days and 14 days of osteogenic induction, cells were washed three times with PBS and fixed in 4% PFA for 15 min. ALP staining was then performed using the Alkaline Phosphatase Stain Kits (Modified Gomori Ca‐CoS Method and Kaplow's/Azo Coupling Method, all purchased from Solarbio, China) according to the manufacturer's instructions.

### 2.11 Immunofluorescence staining

Cells were first cultured on a coverslip (Thermo Fisher, USA). After fixation with 4% PFA, the cells were permeabilized in 0.4% Triton X‐100 for 10 min, and then blocked by 3% BSA for 1 h at room temperature. Primary antibodies (rabbit anti‐rat RUNX2, 1:200, Abcam, ab76956; rabbit anti‐rat p65, 1:200, Abcam, ab32536) were added, and the coverslips were incubated at 4°C overnight. Cells were then washed with PBS and incubated with fluorescent‐labelled secondary antibodies at room temperature for 1 h. The nuclei of cells were counterstained with DAPI for 5 min in the dark. The images were captured under a fluorescence microscope camera (EVOS M5000, Thermo Fisher, USA).

### 2.12 In vitro osteoclast differentiation and TRAP staining

rBMMs were induced to differentiate to osteoclasts by incubating with each complete culture medium (α‐MEM, AnSC‐CM or hBMSC‐CM supplemented with 20 ng/mL M‐CSF and RANKL ranging from 50 to 100 ng/mL). Cells were induced for 5 days and the culture media changed every other day. After 5 days, osteoclasts were stained using a Tartrate Resistant Acid Phosphatase (TRAP) Kit (BestBio, China) following the manufacturer's instructions. Briefly, the cells were fixed in 4% PFA for 15 min, washed with deionized water, and then incubated with TRAP staining solution at 37°C and then counterstained with haematoxylin. The number of TRAP‐positive multinucleated osteoclasts was counted manually.

### 2.13 Quantitative real‐time PCR

Total RNA from each tissue/cell type was isolated using RNAsimple Total RNA Kit (TIANGEN, China) according to the manufacturer's protocol. The specific primers, based on the DNA sequences located in the gene coding regions, were designed using software Primer 5 (Table S1). Glyceraldehyde‐3‐phosphate dehydrogenase (GAPDH) was used as an endogenous control. Total RNA was reverse‐transcribed into cDNA using cDNA Synthesis Kit (Takara, Japan). The SYBR Kit (TRANS, Beijing, China) was used in the quantitative real‐time PCR (qRT‐PCR) assay according to the manufacturer's protocol. Relative expression was calculated using the 2^−ΔΔCT^ method to assess the fold change in expression levels of the target genes.

### 2.14 RNA‐seq

RNA‐sequencing (RNA‐seq) of the healing AB tissues treated with either α‐MEM or AnSC‐CM was done by BGI Life Tech Co. Ltd. (Wuhan, China). The healing tissues in the ABD area were rapidly ground into fine powder in liquid nitrogen using Freezer/Mill 6770 (SPEX CertiPrep Ltd., USA), and total RNA was extracted using Trizol reagent (Qiagen, Hilden, Germany) according to the manufacturer's procedure. RNA quality was confirmed using Bioanalyzer with a minimum RNA integrity number of 7. In total, 0.6 mg RNA was used to construct libraries according to the manufacturer's instructions (Illumina TruSeq Library Preparation Kit v3) and libraries were sequenced using an Illumina HiSeq X Ten at BGI (Shenzhen, China). Sequence reads were deposited in the NCBI Sequence Read Archive (SRA) under accession number PRJNA916673. Gene set enrichment analysis (GSEA) was performed using WebGestalt online tool (http://bioinfo.vanderbilt.edu/webgestalt/).

### 2.15 Statistical analysis

The results are presented as mean ± SEMs. Statistical significance was evaluated using GraphPad Prism 8.0.1 (GraphPad Software, La Jolla, CA) software. The comparisons of single and multiple variables were respectively performed using a one‐way or two‐way ANOVA, and Student's *t*‐test was used to compare two variables. Values were set at *p* < 0.05 for statistical significance.

## 3 RESULTS

### 3.1 AnSC‐CM promoted regeneration of AB tissue in vivo in a rat model

Since AnSCs conferred their regenerative potential primarily in a paracrine manner,^40^, ^41^, ^42^ we investigated whether the AnSC‐CM could exert its therapeutic effects on AB repair in a rat model (Figure 1A) via topical application of the AnSC‐CM‐soaked collagen membrane (colM) to the site of ABD (Figure 1B). The results showed that both AnSC‐CM and hBMSC‐CM groups substantially stimulated regeneration of new bone on the ABD sites and the regenerated bone totally covered the exposed tooth roots on day 21 after treatment; in contrast, the repair process of ABD in the control groups lagged behind and the tooth roots were still clearly seen, evidenced in the reconstructed images using micro‐CT data (Figure 1C,D). Compared with the control groups, both AnSC‐CM and hBMSC‐CM significantly increased bone volume/tissue volume (BV/TV); notably, AnSC‐CM treatment was significantly better on BV/TV than hBMSC‐CM (Figure 1E). The average thickness of bone trabeculae (TbTh) was also significantly increased in the AnSC‐CM group compared with the control groups. However, there was no significant difference with the hBMSC‐CM (Figure 1F). For the mean bone trabecular number (TbN) and bone density (BD), the AnSC‐CM group had the highest values, although there were no significant differences with the other groups (Figure 1G,H).

**FIGURE 2:**
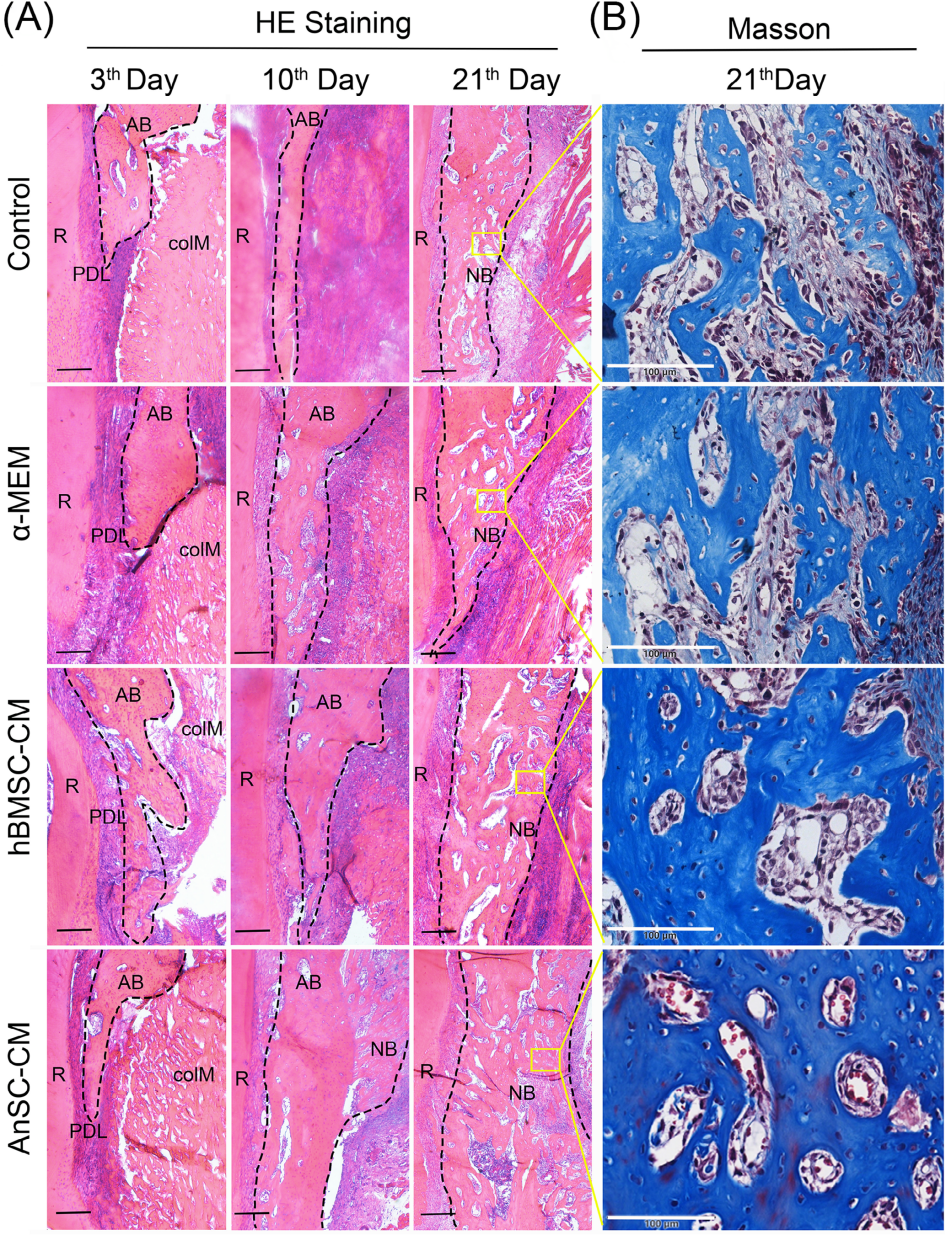
Histological assessment of the effects of AnSC‐CM on repair of ABD. (A) HE staining of the ABD area. No obvious difference could be detected between these groups 3 days after surgery and treatments; and all groups included tooth root (R), periodontal ligament (PDL), ABD and topically‐applied colM, with the infiltration of immune cells. On day 10, NB was seen in the ABD area in the ASC‐CM group. On day 21, NB was detected in the ABD area of every group, but the area of NB in the ASC‐CM group was substantially larger than that of the other groups, and NB had completely covered the ABD area. Scale bar = 250 μm. (B) Masson's Trichrome Staining of the NB from each group on day 21. Note that the NB in the ASC‐CM group was found to be more mature than the other groups, implying that in this group formation of NB was more advanced. Scale bar = 100 μm.

The subsequent histological examination (Figure 2) further confirmed the micro‐CT findings. The AnSC‐CM group regenerated more bone on the ABD site than any other group (Figure 2A) and the regenerated bone in the AnSC‐CM group was more mature than the other groups (Figure 2B). The biosafety examination at the end of the in vivo experiments showed that there were no visible abnormalities in the vital organs (thymus, liver, spleen and kidney, Figure S1), suggesting that AnSC‐CM is safe to use in the rat model. Taken together, these results indicate that topically‐applied AnSC‐CM can effectively stimulate AB tissue regeneration in rats.

### 3.2 AnSC‐CM activated osteogenic pathways but inhibited osteoclastogenic pathways via gene profiling

**FIGURE 3:**
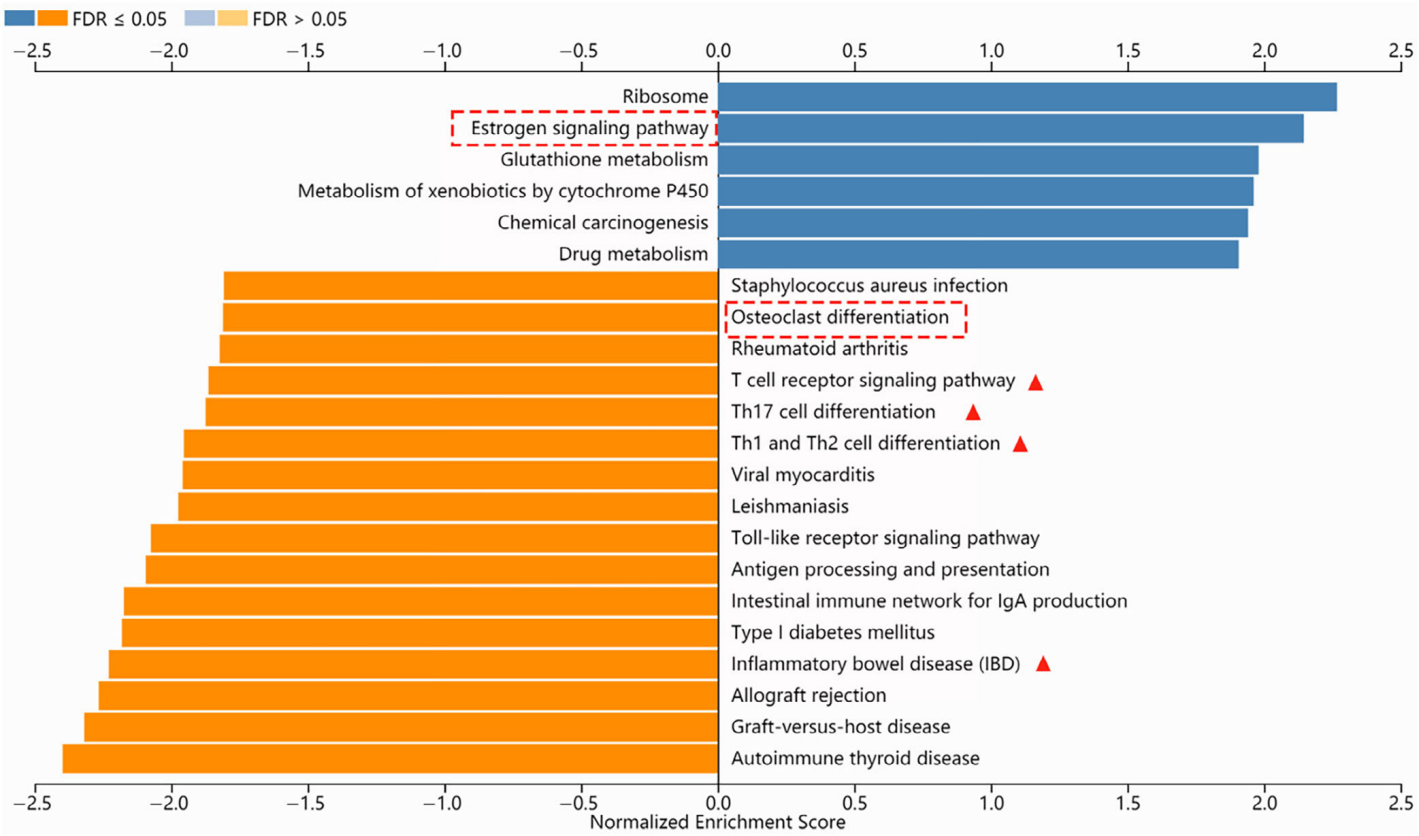
Gene set enrichment analysis (GSEA) for the data obtained from RNA‐seq of the ABD area in the α‐MEM and AnSC‐CM groups. Compared with α‐MEM, AnSC‐CM upregulated the oestrogen signalling pathway (red broken‐line box), but downregulated osteoclast differentiation (red broken‐line box) and some immune responses (red arrowheads). It is known that oestrogen signalling pathway promotes osteogenesis, and inflammation and osteoclastogenesis are detrimental to bone.

To explore the molecular mechanism underlying AnSC‐CM promotion of AB tissue regeneration, RNA‐seq analysis of the regenerated bone and surrounding tissues harvested from the AnSC‐CM and α‐MEM groups was performed 21 days after treatment (Tables S2 and S3). Gene set enrichment analysis (GSEA) was applied to identify the most prominent biological processes, and the results showed that the oestrogen signalling pathway was significantly upregulated, whereas osteoclast differentiation and some immune responses were significantly downregulated in the AnSC‐CM group (Figures 3 and S2). The oestrogen pathway is known to enhance osteogenesis; in contrast, osteoclast differentiation promotes bone destruction.

**FIGURE 4:**
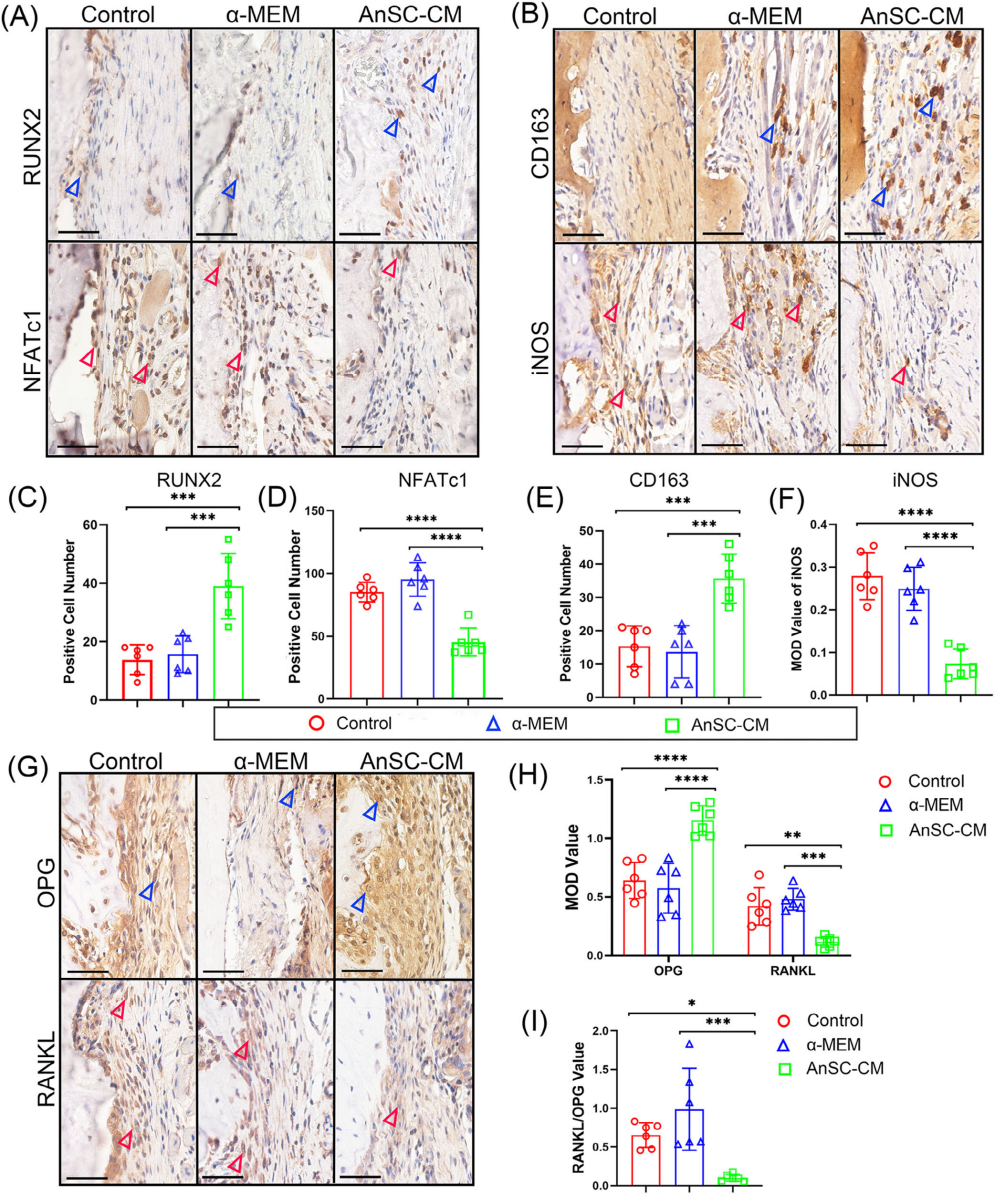
Immunohistochemical (IHC) staining of the tissues in the ABD area sampled on day 21, and comparisons of the AnSC‐CM group with the groups of Control and α‐MEM. (A) IHC staining of RUNX2 (upper) and NFATc1 (lower). Note that AnSC‐CM group had much stronger RUNX2 staining (blue arrowheads); but much weaker NFATc1 staining (red arrowheads). Scale bar = 50 μm. (B) IHC staining of CD163 (upper) and iNOS (lower). Note that AnSC‐CM group had much stronger CD163 staining (blue arrowheads); but much weaker iNOS staining (red arrowheads). Scale bar = 50 μm. (C–F) Quantification of the number of RUNX2^+^, NFATc1^+^ and CD163^+^ cells, and the MOD (mean optical density) value of iNOS, based on the results of (A) and (B). Data is shown as the mean ± SEMs, *n* = 3, six fields were randomly chosen from three replicate of tissue sections for statistical analysis. ***p < 0.001; ****p < 0.0001. (G) IHC staining of OPG (upper) and RANKL (lower). Note that AnSC‐CM group had much stronger OPG staining (blue arrowheads); but much weaker RANKL staining (red arrowheads). Scale bar = 50 μm. (H) The quantification of MOD value of OPG and RANKL. Data is shown as the mean ± SEMs, *n* = 3, six fields were randomly chosen from three replicate of tissue sections for statistical analysis. **p < 0.01; ***p < 0.001; ****p < 0.0001. (I) The ratio of MOD of RANKL/MOD of OPG. *p < 0.05; ***p < 0.001.

To confirm the RNA‐seq findings, expression levels of some key genes for osteogenesis or osteoclastogenesis were evaluated on tissue sections from the ABD site via IHC staining. The results showed that gene expression levels of all three key factors of pro‐osteogenesis, RUNX2 (Figure 4A,C), CD163 (Figure 4B,E) and OPG (Figure 4G,H), were significantly higher in the AnSC‐CM group than in the α‐MEM group. In contrast, gene expression levels of all three key factors of pro‐osteoclastogenesis, NFATc1 (Figure 4A,D), iNOS (Figure 4B,F) and RANKL (Figure 4G,H,I), were significantly lower in the AnSC‐CM group than in the α‐MEM group. Therefore, we conclude that it is highly likely that AnSC‐CM induced AB tissue regeneration achieved through both enhancement of osteogenesis and suppression of osteoclastogenesis.

### 3.3 AnSC‐CM stimulated osteogenic differentiation of rBMSCs in vitro

**FIGURE 5:**
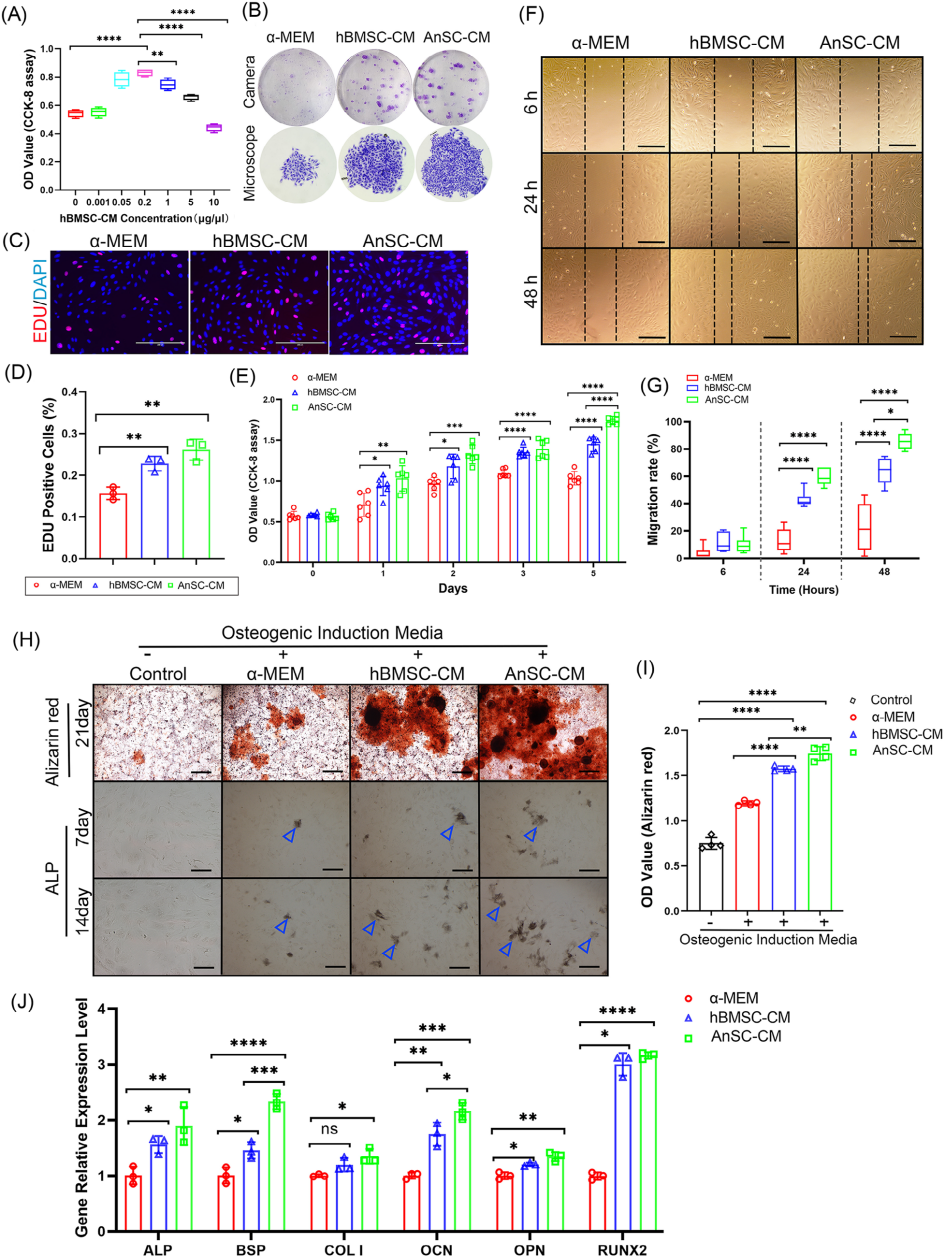
Effects of AnSC‐CM on proliferation, migration and osteogenic differentiation of rBMSCs in vitro. (A) Determination of the optimal concentration of hBMSC‐CM for stimulation of rBMSC proliferation via CCK‐8 assay. Note that 0.2 μg/μL group was the best 24 h after hBMSC‐CM addition. Data is shown as the mean ± SEMs, *n* = 4, **p < 0.01; ****p < 0.0001. (B) Images of colony‐forming units (CFUs; camera; upper) and a single CFU (microscope; lower) in the different groups on day 14. Note that AnSC‐CM group had the highest number of units and the largest unit in size. (C) Mitogenic effects of AnSC‐CM on rBMSCs via EDU incorporation assays. Red fluorescence: EDU‐positive cells. Scale bar = 200 μm. (D) Percentage of EDU positive cells. Note that AnSC‐CM group had higher percentage of EDU‐positive cells than hBMSC‐CM, although no statistical difference detected. Data is shown as the mean ± SEMs, *n* = 3, **p < 0.01. (E) Proliferation rates of rBMSCs in different groups via CCK‐8 assay during 5‐day incubation. Note that on day 5, AnSC‐CM group was significantly higher than hBMSC‐CM group. Data is shown as the mean ± SEMs, *n* = 6, *p < 0.05; **p < 0.01; ***p < 0.001; ****p < 0.0001. (F) Representative images of scratch assays of rBMSCs incubated in the different media. Note that in the AnSC‐CM medium, rBMSCs had almost completed filling the wounded spaces via migration 48 h after scratch. Scale bar = 300 μm. (G) Quantification of migration rates of rBMSCs in the different groups, based on the results of (F). Data is shown as the mean ± SEMs, *n* = 6, *p < 0.05; ****p < 0.0001. (H) Representative images of mineralized nodules (red colour) formed by rBMSCs, Alizarin Red staining (upper, scale bar = 400 μm) on day 21 in the osteogenic induction media, and representative images of ALP staining (blue arrowheads; lower; Scale bar = 150 μm) on days 7 and 14 in the osteogenic induction media. Note that AnSC‐CM group had overall more and larger mineralized nodules and higher expression levels of ALP than the other groups. (I) Mineralization quantification based on the results of (H). Note that AnSC‐CM group had the highest mineralization value than the other groups including hBMSC‐CM group. Data is shown as the mean ± SEMs, *n* = 4, **p < 0.01; ****p < 0.0001. (J) Relative expression levels of osteogenic genes on day 7 in the osteogenic induction media, including ALP, BSP, COL I, OCN, OPN and Runx2. Overall, ASC‐CM group had stronger effects on the expressions of these genes. Data is shown as the mean ± SEMs, *n* = 3, *p < 0.05; **p < 0.01; ***p < 0.001; ****p < 0.0001.

Tissue repair/regeneration after injury are reported to involve the selective recruitment of endogenous BMSCs.^43^ We sought to explore whether the effects of AnSC‐CM on AB regeneration in rats were achieved partially via stimulating proliferation, migration and homing of endogenous BMSCs. First, we established the optimal concentration of hBMSC‐CM on proliferation rate of the rBMSCs in vitro, that is, 0.2 μg/μL (Figure 5A); then we used this concentration for the AnSC‐CM group in comparison with the hBMSC‐CM group for the following experiments. In the colony‐forming unit assay, AnSC‐CM treatment promoted the rBMSCs to form highest number of units and the largest in size, compared with the other groups (Figure 5B). In the EDU incorporation assay, the highest number of EDU^+^ cells of rBMSCs were found in the AnSC‐CM group, although no statistical significance was detected when compared with hBMSC‐CM group (Figure 5C,D). In the proliferation assay, the rates of rBMSCs in both AnSC‐CM and hBMSC‐CM groups were significantly higher than that in the α‐MEM group between days 1 and 5 in culture; notably, on day 5, the rate in AnSC‐CM group was significantly higher than that of the hBMSC‐CM group (Figure 5E).

In the migration assay, rBMSCs in the AnSC‐CM group showed the highest migration rate among all three groups and almost completely filled the wounded space 48 h after the scratch; notably, the migration rate of rBMSCs in the AnSC‐CM group was significantly higher than that in the hBMSC‐CM group (Figure 5F,G).

We next examined whether AnSC‐CM could potentiate osteogenesis of rBMSCs in vitro. In the osteogenic induction assay, rBMSCs in the AnSC‐CM group formed more and larger mineralized nodules and had higher expression levels of ALP (a marker for an early stage of mineralization)^44^ than the other groups (Figure 5H); notably, rBMSCs in the AnSC‐CM group was significantly more mineralized than that in the hBMSC‐CM group (Figure 5I). In the osteogenic gene expression assay, expression levels of ALP, BSP, OCN, OPN and RUNX2 of the rBMSCs in both AnSC‐CM and hBMSC‐CM groups were overall significantly higher than those in the α‐MEM group. Notably, expression levels of BSP and OCN in the AnSC‐CM group were significantly higher than those in the hBMSC‐CM group (Figure 5J). Overall, at the cellular, molecular and functional levels, AnSC‐CM exhibited a strong potential to drive endogenous rBMSCs to home and differentiate toward osteogenic lineage cells, and this potential was even stronger than hBMSC‐CM; this explains why AnSC‐CM had better healing power for ABD in the rat model.

### 3.4 AnSC‐CM promoted osteogenic differentiation of hPDLSCs in vitro

**FIGURE 6:**
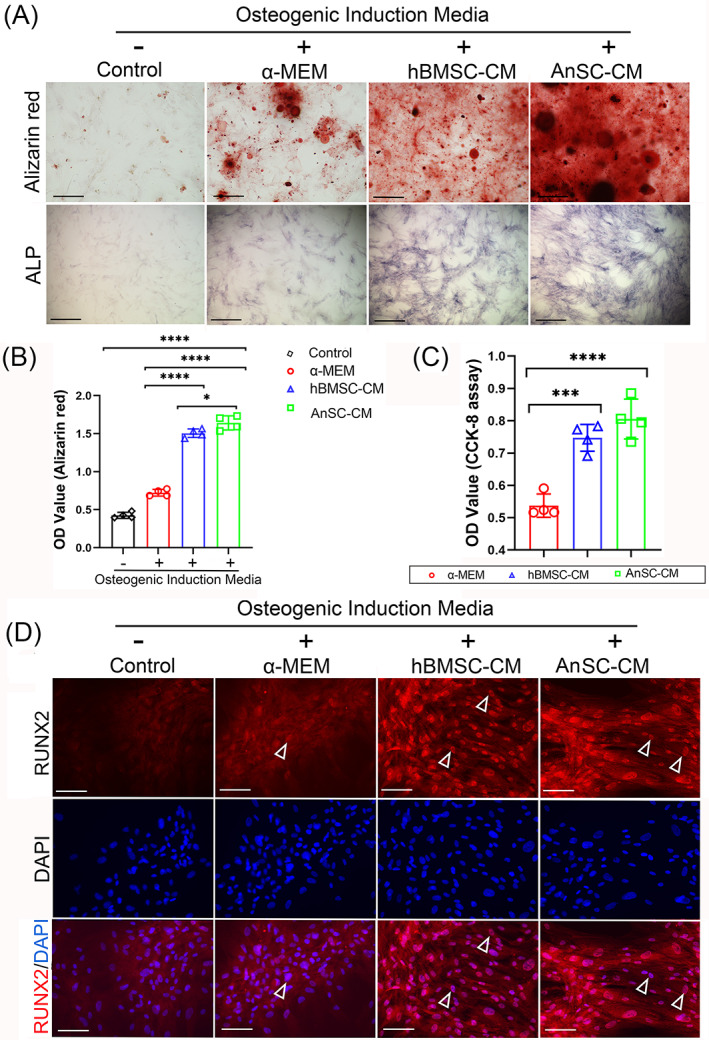
Effects of AnSC‐CM on proliferation and osteogenic differentiation of hPDLSCs in vitro. Representative images of mineralized nodules (red colour) formed by hPDLSCs, Alizarin Red staining (upper) on day 14 in the osteogenic induction media, and representative images of ALP staining (lower) on day 7 in the osteogenic induction media. Scale bar = 150 μm. Note that AnSC‐CM group was the most mineralized and the highest ALP expression group. (B) Mineralization quantification, based on the results of (A). Note that AnSC‐CM group was the highest in all the groups and even significantly higher than hBMSC‐CM group. Data is shown as the mean ± SEMs, *n* = 4, *p < 0.05; ****p < 0.0001. (C) Mitogenic effects of AnSC‐CM on hPDLSCs in the different groups via CCK‐8 assay. Note that AnSC‐CM had the strongest mitogenic effects than the other groups, although not statistically different with the hBMSC‐CM group. Data is shown as the mean ± SEMs, *n* = 4, ***p < 0.001; ****p < 0.0001. (D) Representative images of RUNX2 expression via IHC staining (red fluorescence) and the nuclei were stained by DAPI (blue). Note that RUNX2 were highly expressed in both hBMSC‐CM and AnSC‐CM groups. Scale bar = 150 μm.

The ultimate goal of the study is to apply AnSC‐CM to repair ABD in the clinical setting; thus it is important to know whether AnSC‐CM could play a role in stimulating resident cells to repair the defects in human as effectively as in rats. In the experiment, we selected hPDL stem cells (hPDLSCs) as PDL is not only an integral part of periodontal tissue, but hPDLSCs can differentiate into multiple cell types to participate in repair of defects.^45^ Our results showed that in the osteogenic induction assay, hPDLSCs in the AnSC‐CM group formed more and larger mineralized nodules on day 14 and had higher expression levels of ALP on day 7 than the other groups (Figure 6A). Notably, hPDLSCs in the AnSC‐CM group were significantly more mineralized than those in the hBMSC‐CM group (Figure 6B). In the proliferation assay, AnSC‐CM showed the highest mitogenic effects on hPDLSCs compared with the other groups, although it was not statistically different from the hBMSC‐CM group (Figure 6C). In the osteogenic gene expression assay, expression levels of RUNX2 in both hBMSC‐CM and AnSC‐CM groups were much higher than in the control groups on day 7 after osteogenic induction (Figure 6D), although no quantitative comparison in the expression level between the hBMSC‐CM and AnSC‐CM groups was carried out. Overall, the effects of AnSC‐CM on the osteogenesis of hPDLSCs were comparable to those on rBMSCs, indicating that AnSC‐CM would be expected to be equally effective if applied in the clinical setting. Nonetheless, we are fully aware of that some major issues must be properly addressed before this translation can take place, including cross‐species safety hazards, ethical and legal issues, and the composition of AnSC‐CM and so on.

### 3.5 AnSC‐CM exerted an anti‐inflammatory role via inhibition of rBMM polarization toward M1 in vitro

**FIGURE 7:**
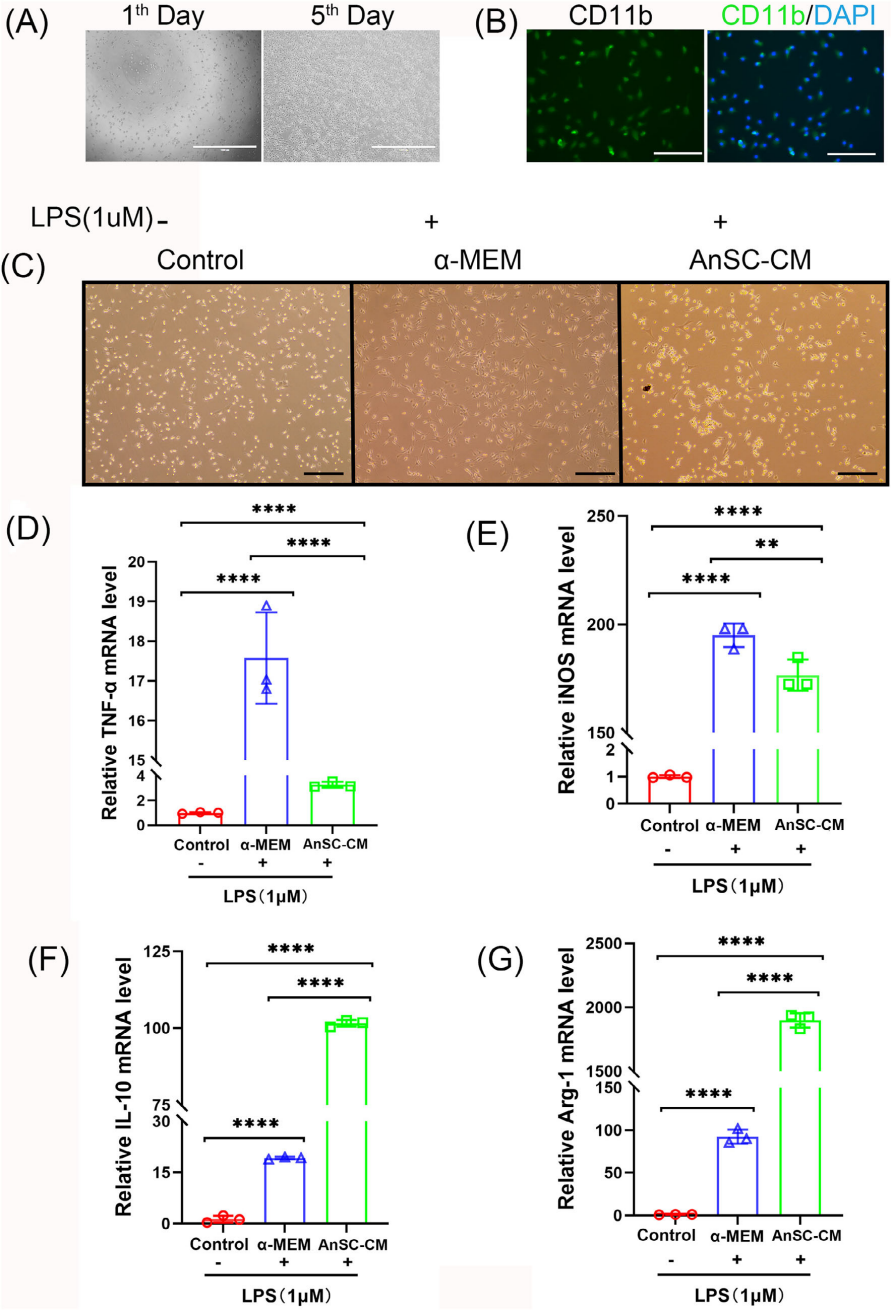
Effects of AnSC‐CM on inflammation of rBMMs induced by LPS in vitro. (A) Characterization of the freshly isolated rat BMMs (rBMMs) via treatment with 20 ng/mL M‐CSF. Cell viability was recorded on days 1 and 5 (Scale bar = 1000 μm). (B) High expression level of CD11b on rBMMs was detected (Scale bar = 300 μm), thus confirmed the nature of the isolated cells as rBMMs. (C) Polarized macrophages displayed in different morphologies. Note that rBMMs in the α‐MEM medium exhibited spindle shape and possessed elongated pseudopodia (M1 phenotype) under LPS‐induction; whereas in the AnSC‐CM medium, rBMMs were bright and round, with much fewer protruding pseudopodia (M0 phenotype). Scale bar = 150 μm. (D–G) mRNA expression levels of TNF‐α, iNOS, IL‐10 and Arg‐1 in the different groups after LPS induction. Note that AnSC‐CM significantly downregulated pro‐inflammatory factors TNFα and iNOS, but significantly upregulated anti‐inflammatory factors IL10 and Arg‐1, compared with the control groups. Data is shown as the mean ± SEMs, *n* = 3, **p < 0.01; ****p < 0.0001.

AB destruction is normally caused by periodontitis, and here we sought to determine whether AnSC‐CM could play an anti‐inflammatory role via immunomodulation, besides stimulation of osteogenesis, using freshly isolated rBMMs. First, we demonstrated that our rBMMs were viable (Figure 7A) and expressed the rBMM marker gene, CD11b (Figure 7B). Subsequently, the rBMMs were treated with LPS to induce them to polarize toward M1, the pro‐inflammatory type of macrophage. The results showed that rBMMs in the α‐MEM + LPS medium exhibited spindle shape and possessed elongated pseudopodia (M1 phenotype), whereas, in the AnSC‐CM + LPS medium, rBMMs were bright and round, with much fewer protruding pseudopodia, which was more similar to the phenotype of the control (M0 phenotype; non‐induced medium; Figure 7C). Therefore, AnSC‐CM effectively inhibited rBMMs polarization toward M1. Next, we studied the effects of AnSC‐CM on the expressions of pro‐inflammatory and anti‐inflammatory genes of the LPS‐induced rBMMs. The results showed that AnSC‐CM significantly downregulated expressions of pro‐inflammatory genes TNF‐α (Figure 7D) and iNOS (Figure 7E), but significantly upregulated expressions of anti‐inflammatory genes IL‐10 (Figure 7F) and Arg‐1(Figure 7G), compared with the α‐MEM group. Notably, in the AnSC‐CM group, the expression level of TNF‐α was 5 times lower (Figure 7D); in contrast, IL‐10 was 5 times higher (Figure 7F), and Arg‐1 was nearly 20 times higher (Figure 7G) than those in the α‐MEM group. These results indicate that AnSC‐CM has strong anti‐inflammatory potential through down‐regulating the expression of pro‐inflammatory factors and up‐regulating the expression of anti‐inflammatory factors; and downregulating BMMs polarization toward M1 type.

### 3.6 AnSC‐CM inhibited osteoclast differentiation of rBMMs in vitro

**FIGURE 8:**
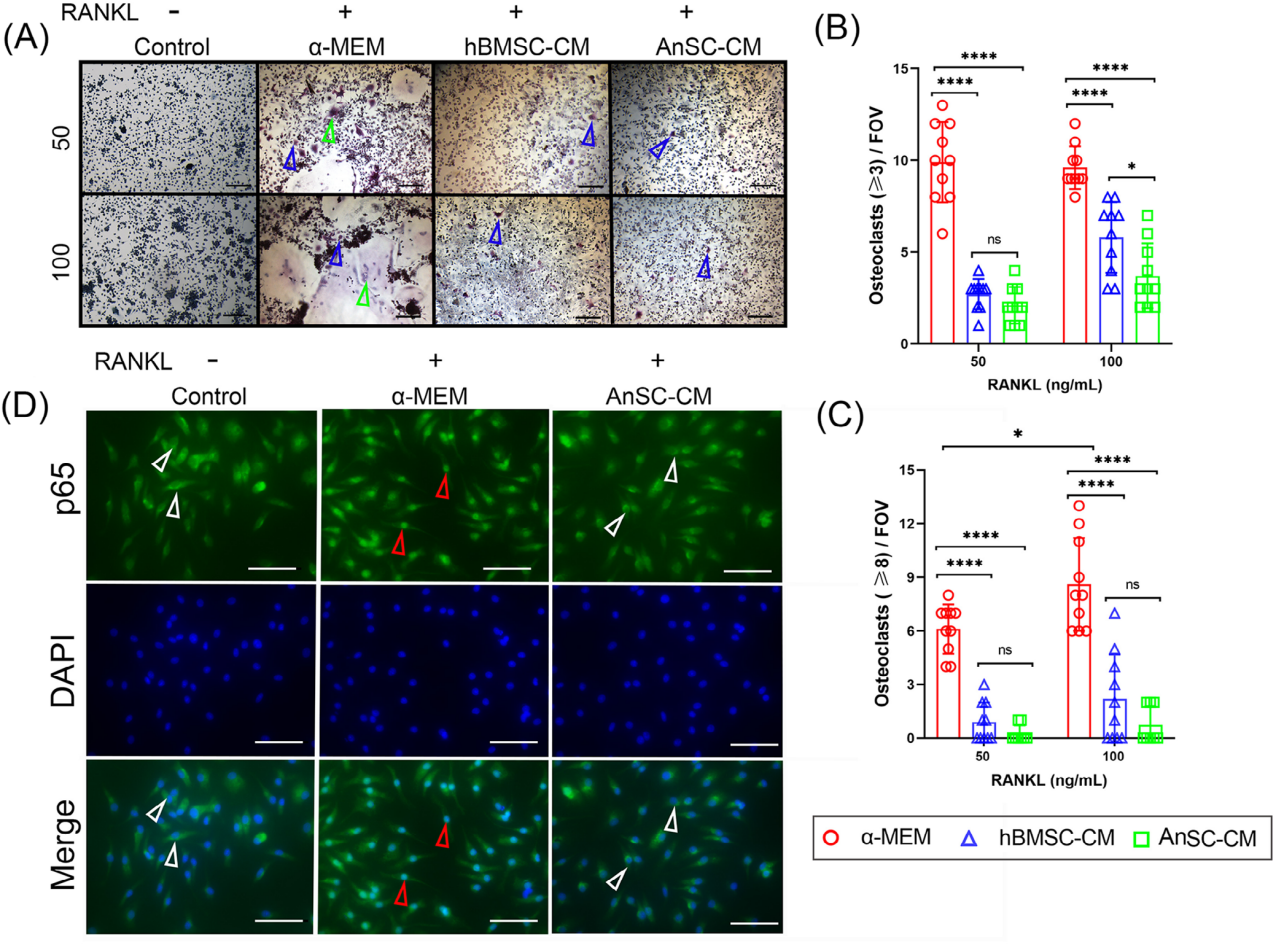
Effects of AnSC‐CM on osteoclast differentiation of rBMMs. Osteoclast differentiation (multinucleated, arrowheads) of the rBMMs 5 day after induction with RANKL. Note that both AnSC‐CM and hBMSC‐CM groups significantly reduced osteoclast differentiation compared with the control group. Blue arrowheads: osteoclasts with ≥3 nuclei; Green arrowheads: osteoclasts with ≥8 nuclei. Scale bar = 400 μm. (B,C) Quantification of number of the TRAP‐positive osteoclasts with ≥3 nuclei (B) and with ≥8 nuclei (C). Note that at the dose of 100 mg/mL RANKL, AnSC‐CM more significantly downregulated osteoclast formation than hBMSC‐CM. *N* = 3, Ten fields of view (FOV) were randomly chosen from three replicates for statistical analysis. Data is shown as the mean ± SEMs. *p < 0.05; ****p < 0.0001. (D) Representative images of p65 expression via IHC staining. Note that RANKL treatment induced p65 translocation from cytoplasm to nuclei (red arrowheads) in the α‐MEM group, but failed to do so in the AnSC‐CM group where p65 remained in the cytoplasm (white arrowheads). Scale bar = 150 μm.

The immune system participates in bone destruction mainly through initiating inflammation as well as osteoclastogenesis,^46^ so we asked whether AnSC‐CM was also able to suppress osteoclastogenesis in addition to its inhibitory effect on inflammation. Two concentrations of RANKL, 50 and 100 ng/mL were used in the experiment to induce differentiation of rBMMs toward osteoclasts. The results showed that TRAP‐positive multinucleated osteoclasts in both the AnSC‐CM and hBMSC‐CM groups were visibly fewer than those in the α‐MEM group (Figure 8A). Notably, at a concentration of 100 ng/mL RANKL, osteoclasts (≥3 nuclei) in the AnSC‐CM group were significantly fewer than those in the hBMSC‐CM group (Figure 8B); and osteoclasts (≥8 nuclei) in the AnSC‐CM group were barely detectable (Figure 8C). Therefore, AnSC‐CM has the ability to effectively suppress osteoclastogenesis.

To explore the molecular mechanism underlying this suppression, we again used RANKL to treat rBMMs for osteoclast differentiation. The treatment effectively induced translocation of p65 from the cytoplasm to nucleus, a hallmark of NF‐κB pathway activation, in the α‐MEM group, whereas, in the AnSC‐CM group, p65 translocation was not detectable (Figure 8D), indicating that AnSC‐CM effectively suppressed the p65 translocation process and thus inactivated NF‐κB transduction pathway. Therefore, we conclude that it is likely that AnSC‐CM suppressed osteoclast differentiation through inhibition of activation of NF‐κB transduction pathway.

## 4 DISCUSSION

To the best of our knowledge, this is the first time that AnSC‐CM was used to treat surgically created intrabony ABD in a rat model. The results convincingly demonstrated that AnSC‐CM effectively induced regeneration of AB tissue, which properly filled in the ABD; in some key aspects, AnSC‐CM was significantly better than hBMSC‐CM, such as in the BV/TV parameter and degree of bone maturity. The results of the present study support the hypothesis that the effects of AnSC‐CM on bone regeneration were achieved through both stimulation of osteogenesis and inhibition of osteoclastogenesis. These include activation of the oestrogen signalling pathway and inhibition of osteoclast differentiation and immune response pathways; upregulation of osteogenic factors and downregulation of osteoclastic differentiation factors; stimulation of the proliferation, migration and differentiation of resident MSCs toward osteogenic lineages; modulation of macrophage polarization toward the M2 phenotype; and suppression of osteoclastogenesis. In addition, we believe that bringing the concept of neural crest lineage into the field of oral health would facilitate the identification of new and novel types of MSCs for more effective treatment. Overall, AnSC‐CM may be a type of desirable MSC‐CM for effectively repairing damage of periodontal tissues in the dental clinical setting.

### 4.1 Promotion of osteogenesis via homing of endogenous MSCs

It is known that MSC‐CM can effectively promote periodontal tissue regeneration because the media contributes to the mobilization, proliferation and differentiation into osteoblasts of endogenous MSCs, finally leading to the regeneration of AB and other periodontal tissue types.^47^ In the present study we investigated the efficacy of AnSC‐CM by applying AnSC‐CM‐soaked collagen membranes to surgically created ABD and explored the underlying mechanism in vitro using both rBMSCs and rBMMs.

The mobilization/homing and proliferation of resident MSCs for tissue regeneration have attracted attention in the field of regenerative medicine, and these have been considered critical for MSC‐CM to effectively repair the ABD.^48^, ^49^ Resident MSCs can be directed to the defects to exert regenerative functions, which is similar to or more effective than the direct use of transplanted foreign stem cells.^7^ The effects of MSC‐CM on regeneration of AB tissue have been considered via acceleration of the mobilization and proliferation of endogenous MSCs and endothelial cells.^50^, ^51^ In the present study, we found that, compared with the control in vitro, AnSC‐CM significantly stimulated proliferation of rBMSCs through assays of CCK8, colony‐forming unit and EDU incorporation, and significantly promoted migration of rBMSCs through a scratch assay. Notably, some of these parameters were significantly better than those in the hBMSC‐CM group. These include proliferation rate, colony‐forming unit and migration rate (Figure 5A–G). Thus, treatment of AnSC‐CM effectively mobilized endogenous MSCs and stimulated their proliferation.

The ultimate test of the impact of AnSC‐CM on AB tissue regeneration would be to see whether it could facilitate the homing of endogenous MSCs to the injured site and the differentiation of the mobilized MSCs into bone lineage cells to form an adequate amount of bony tissue that is properly mineralized. In our recent studies, AnSCs initiated antler regeneration though activating osteogenesis‐related pathways and upregulating bone formation‐related genes (e.g., RUNX2 and BMP4; Figure S3).^36^ In the present study, expression levels of osteogenic marker genes both in vivo (OPG, RUNX2, CD163) in a rat model and in vitro (ALP, OCN, COL 1, OPN and Runx2) in a culture system for rBMSCs were significantly higher in the AnSC‐CM group than in the control group. Notably, expression levels of some genes were even significantly higher than in the hBMSC‐CM group. These indicate that AnSC‐CM effectively stimulated endogenous MSCs to differentiate toward osteogenic lineage cells. Mineralization of bone nodules was assessed using Alizarin Red staining, and the degree of mineralization of the nodules using both rBMSCs and hPDLSCs were found to be significantly higher in the AnSC‐CM group than in the hBMSC‐CM group. In addition, the AnSC‐CM simulated more bone tissue regeneration on the ABD site than any other treatments, indicating that AnSC‐CM has stronger osteogenic potential. The regenerated bone tissue in the AnSC‐CM group was more mature than the other groups, indicating bone formation in the AnSC‐CM group was more advanced. Overall, we conclude that AnSC‐CM must have promoted AB tissue regeneration through stimulation of the mobilization and proliferation of the endogenous MSCs, and differentiation of these lodged MSCs toward bone lineage cells.

### 4.2 Inhibition of osteoclastogenesis via immunomodulation

AnSC‐CM enhanced AB tissue regeneration in an adult immunocompetent rat model, indicating that it effectively modulated the host immune system, which is consistent with previous studies using other MSC‐CM sources.^52^, ^53^ For example, BMSCs locally injected into mouse periodontal defects exert anti‐inflammatory and immunomodulatory effects at the target site and contribute to the regeneration of new tissue.^9^, ^10^ Further in our study, RNA‐seq analysis revealed that AnSC‐CM significantly downregulated some immune responses in vivo and the expression level of TNF‐α in vitro. It is known that TNF‐α is a potent proinflammatory cytokine and involved in inflammation and immune responses.^13^ Furthermore, there was no inflammatory reaction such as redness, swelling, or ulceration around the site of AnSC‐CM application; and there were no visible morphological changes found in the vital organs (Figure S1), which is consistent with recent reports of the topical application of various CMs.^32^, ^54^ Therefore, suppression of immune responses and the expression level of TNF‐α in the AnSC‐CM group would have partially explained why this CM did not elicit immune rejection.

In the process of tissue repair, the crosstalk between MSCs and immune cells is crucial for achieving optimal regeneration.^55^ Macrophages are the most important players from immune side, and commonly divided into classically activated macrophage (M1, pro‐inflammatory type) and alternatively activated macrophage (M2, anti‐inflammatory type).^56^, ^57^ According to recent studies, macrophage polarization toward the anti‐inflammatory type M2 is always correlated with better regeneration outcomes.^58^, ^59^ Therefore, effective repair of ABD by application of AnSC‐CM may have been partially through its paracrine effect on macrophage polarization toward M2. Indeed, AnSC‐CM treatment significantly elevated expression levels of CD163 in the regenerated periodontal tissue and IL‐10 in the rBMM cells (nonactivated macrophages) in vitro. However, it lowered the expression levels of TNF‐α and iNOS both in vivo and in vitro. It is known that CD163 and IL‐10 are biomarkers of M2^60^ while TNF‐α and iNOS are biomarkers of M1.^61^ Therefore, the application of AnSC‐CM effectively promoted M2 macrophage polarization and at the same time suppressed M1 macrophage activation, the consequence of which is the reduced ratio of M1/M2.

It is known that destruction of periodontal tissues is mainly caused by the host immune response to inflammation,^62^ polarization of infiltrated immune cells and activation of osteoclast differentiation.^63^ Osteoclasts are giant, multi‐nucleated, and bone‐resorbing cells. Receptor activator of nuclear factor‐kB ligand (RANKL) is the principal factor involved in osteoclast differentiation, activation, and survival. RANKL, either independently or synergistically with Lipopolysaccharide (LPS, from Gram‐negative bacteria), can regulate osteoclastogenesis.^64^ RANKL promotes the fusion of mononuclear precursors to form multinucleated osteoclasts.^65^ Osteoclastogenesis was completely abrogated upon addition of osteoprotegerin (OPG), the decoy receptor of RANKL.^64^ Convincingly, in the present study, application of AnSC‐CM significantly decreased RANKL expression and increased OPG expression in the regenerated AB tissue in vivo, and significantly reduced the number of osteoclasts formed in the rBMMs in vitro, evidenced by staining with Tartrate‐resistant acid phosphatase (TRAP), a marker for mature osteoclasts.^65^ Therefore, the effects of AnSC‐CM treatment on the inhibition of osteoclastogenesis and regeneration of AB tissue may have been achieved through the aforementioned pathways.

Freshly isolated BMMs from long bones of mice could not differentiate into osteoclasts when stimulated with LPS alone.^66^ Osteoclastogenesis by LPS was reinstated when BMMs were pretreated with RANKL. In contrast, LPS pretreated BMMs that were then stimulated by RANKL failed to undergo osteoclast differentiation.^66^ This is thought to be due to the ability of LPS to downregulate expression of RANKL‐induced nuclear factor of activated T‐cells cytoplasmic 1 (NFATc1), a master regulator of osteoclastogenesis. Therefore, factors that can downregulate NFATc1 would be able to effectively suppress osteoclastogenesis. In our study, expression of NFATc1 in the regenerated AB tissue was significantly down‐regulated, which would thus result in impaired osteoclastogenesis.

The role of M1 and M2 macrophages in the modulation of osteoclastogenesis and AB resorption has been elucidated recently. Generally, the ratio of M1/M2 macrophages is positively correlated with the progression of periodontal diseases.^67^, ^68^ M2 activation reduces TNF‐α secretion in RAW 264.7 cells and decreases AB resorption in a murine periodontitis model. Fewer osteoclasts were observed in the group with the activated M2 profile compared with the control group.^69^ In the present study, AnSC‐CM treatment not only significantly decreased the ratios of M1/M2 (see marker for these phenotypes), but also significantly downregulated TNF‐α secretion by rBMMs. Therefore, the reduction of osteoclastogenesis in the AnSC‐CM group in the present study would be attributed partially to the decrease in the ratio of M1/M2 and expression level of TNF‐α.

### 4.3 Better healing power via tissue/cell compatibility (neural crest‐derived vs. mesoderm‐derived)?

Why AnSC‐CM functioned better than hBMSC‐CM at stimulation of AB tissue regeneration in the present study can only be subject to speculation. One possible explanation is the embryonic origin: while AnSCs are neural crest‐derived,^28^, ^29^, ^30^ BMSCs are mesoderm‐derived.^7^ Given that all periodontal tissues are of neural crest‐derivation,^16^, ^17^ for the reason of tissue/cell compatibility, AnSCs should have advantages over BMSCs. Indeed, Leucht et al. found an interesting phenomenon that injured bones preferentially heal using cells of the same embryonic origin.^19^ In the present study, cells (cell‐CM) from the same embryonic origin (neural crest‐derived AnSC‐CM on neural crest‐derived rABD) showed better healing effects than cells (cell‐CM) from the same species (human‐derived BMSC‐CM on human PDLSCs). However, the mechanism underlying this phenomenon is not known. Despite being similar in phenotype and immunotype, Mrozik et al. identified the differential protein expression pattern between PDLSCs and BMSCs via proteomic approaches and suggested that this differential protein expression could explain the difference in effects on periodontal wound healing: the former is of neural crest origin and the latter of mesoderm origin.^70^ Very recently, Zou et al. took the transcriptomic approach and revealed 589 genes differentially expressed between hPDLSCs (neural crest‐derived) and BMSCs (mesoderm‐derived), including eightfold higher levels of BMP4 in the hPDLSCs.^8^ In the present study, we found that AnSCs also highly expressed BMP4 expression (Figure S3F). In addition, a recent report showed that AnSC had a faster proliferation rate than hBMSC.^40^ Therefore, CM from the former is likely to stimulate a faster regeneration than the hBMSC‐CM. Further identification of the effective molecules from the neural crest‐derived CMs would greatly facilitate the development of better healing drugs that are cell‐free, composition‐clear, ready‐to‐use and more amenable to reformulation. We have been continuously working on the identification of more effective molecules on treating ABD along this line. In addition, introduction of the concept of embryonic origin to the field of dental research would open up a new avenue to search for more kinds of neural crest‐derived MSCs for more effective treatment of periodontal tissue defects in the dental clinic setting.

## 5 CONCLUSION

**FIGURE 9:**
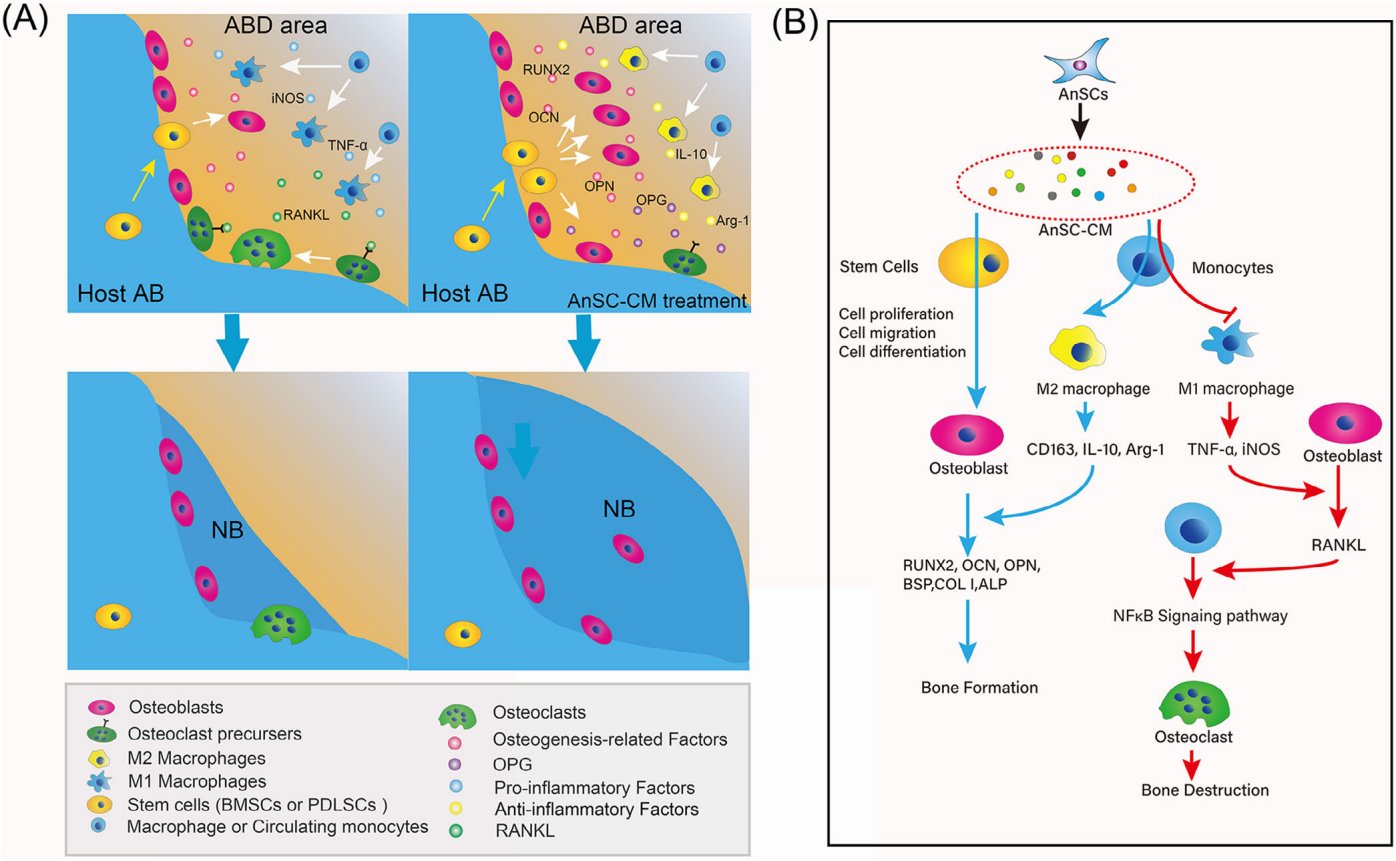
Schematic illustration of the molecular mechanism underlying the effective treatment of the ABD by AnSC‐CM. (A) Surgical creation of the ABD promoted the homing of endogenous MSCs, PDLSCs and immune cells to the injured area, and induced polarization of circulating monocytes toward M1 type (pro‐inflammatory) and osteoclastogenesis, resulted in bone resorption. Application of AnSC‐CM on the defects modulated the homing immune cells and coaxed them toward M2 (anti‐inflammatory) polarization. At the same time, AnSC‐CM promoted the homing MSCs to differentiate into osteoblasts and resulted in more AB tissue regeneration than the control. (B) AnSC‐CM treatment to the ABD area, on one hand, induced homing MSCs to different into osteoblasts and elevated expression levels of osteogenic genes for the promotion of new bone tissue formation; on the other hand, induced circulating monocytes to polarize to anti‐inflammatory type M2 macrophage and stimulated expressions of anti‐inflammatory genes to further enhance osteogenesis via osteoblast differentiation; and at the same time, inhibition of polarization of pro‐inflammatory M1 macrophage and expressions of pro‐inflammation factors effectively suppressed osteoclastogenesis via inactivation of NFκB signalling pathway. Overall, the effects of AnSC‐CM on AB tissue regeneration were achieved through both stimulation of osteogenesis and inhibition of osteoclastogenesis.

Based on the overall results of the present study, our working hypothesis is that, in the periodontitis environment, LPS released by the bacteria interacts with TLR4 on the recruited endogenous monocytes to promote the polarization of these cells toward pro‐inflammatory macrophages (M1) and stimulate M1 to secrete pro‐inflammatory cytokines such as TNF‐α, which then stimulates receptor activator of nuclear factor kappa‐B ligand (RANKL) expression on osteoblasts. RANKL produced by osteoblasts binds receptor activator of nuclear factor kappa‐B (NF‐κB), a receptor expressed in osteoclast precursor cells, and the complex then activates osteoclast differentiation and bone resorption. Application of AnSC‐CM coaxes the monocytes to polarize to the anti‐inflammatory M2 type, rather than the M1 type, and the M2 type expresses anti‐inflammatory cytokines to enhance osteoblast differentiation. At the same time, AnSC‐CM induces the lodged MSCs to differentiate to osteoblast lineage cells and express osteogenic factors, which then promotes osteogenesis and new AB tissue formation (Figure 9).

## AUTHOR CONTRIBUTIONS

Qianqian Guo and Chunyi Li conceived and designed the experiments, performed part of experiments; Qianqian Guo, Chunyi Li and Rui Du drafted the article and provided funding supports; Junjun Zheng performed the bioinformatics and statistical analyses; Zhongming Han, Hongbing Lin, Haiping Zhao and Zhen Wang carried out the animal experiments and part of in vitro experiments; Jing Ren and Jingjie Zhai performed the histology. All authors read and approved the final manuscript.

## CONFLICT OF INTEREST STATEMENT

The authors declare that there is no conflict of interests.

## Supporting information


**Figure S1.** Morphological examination of vital organs including thymus, liver, kidney and spleen. There were no visible abnormities detected in the thymus, liver, spleen and kidney from the AnSC‐CM treated rats.

**Figure S2.** Gene set enrichment analysis (GSEA) of RNA‐seq data. (A) Oestrogen signalling pathway was activated. (B) Osteoclast differentiation, some immune responses were inhibited. (C) Th17 cell differentiation was inhibited. (D) Th1 and Th2 cell differentiation was inhibited. Note that all these were inhibited in the AnSC‐CM group compared with the α‐MEM group. FDR, false discovery rate (adjusted *p* value).

**Figure S3.** Up‐regulated DEGs in the activated AnSCs are mainly osteogenesis‐related (A) GO analyses of up‐regulated DEGs in the activated antler stem cells (AnSCs). (B) Module analysis of all up‐regulated DEGs. Four clusters were screened with a cut‐off k‐score = 5 depending on the MCODE scoring system. (C) GO analyses of DEGs in the cluster 3. (D) Protein–protein interaction networks for genes in cluster 3. Note that RUNX2 was located at the centre of the network. (E,F) Immunohistochemical staining (IHC) of RUNX2 (E) and BMP4 (F) in the AnSCs. RUNX2 positive staining was mainly localized in the nucleus, while BMP4 positive staining was mainly located in the cytoplasm. Scale bar = 100 μm.

**Table S1.** Primers used for qRT‐PCR.
**Table S2.** Summary of transcriptome sequencing data.
**Table S3.** Alignment rates of effective RNA‐seq sequences on the reference genome.

## Data Availability

The authors confirmed that all data needed to evaluate the conclusions in the paper are presented in the paper and/or the Supplementary Materials. Additional data related to this paper can be requested from the authors.
