# Supplementary figures and images for: Conditioned media of deer antler stem cells accelerate regeneration of alveolar bone defects in rats
Source: Cell Prolif. 2023 Mar 16;56(5):e13454. doi: 10.1111/cpr.13454 (PMC10212714; doi:10.1111/cpr.13454)

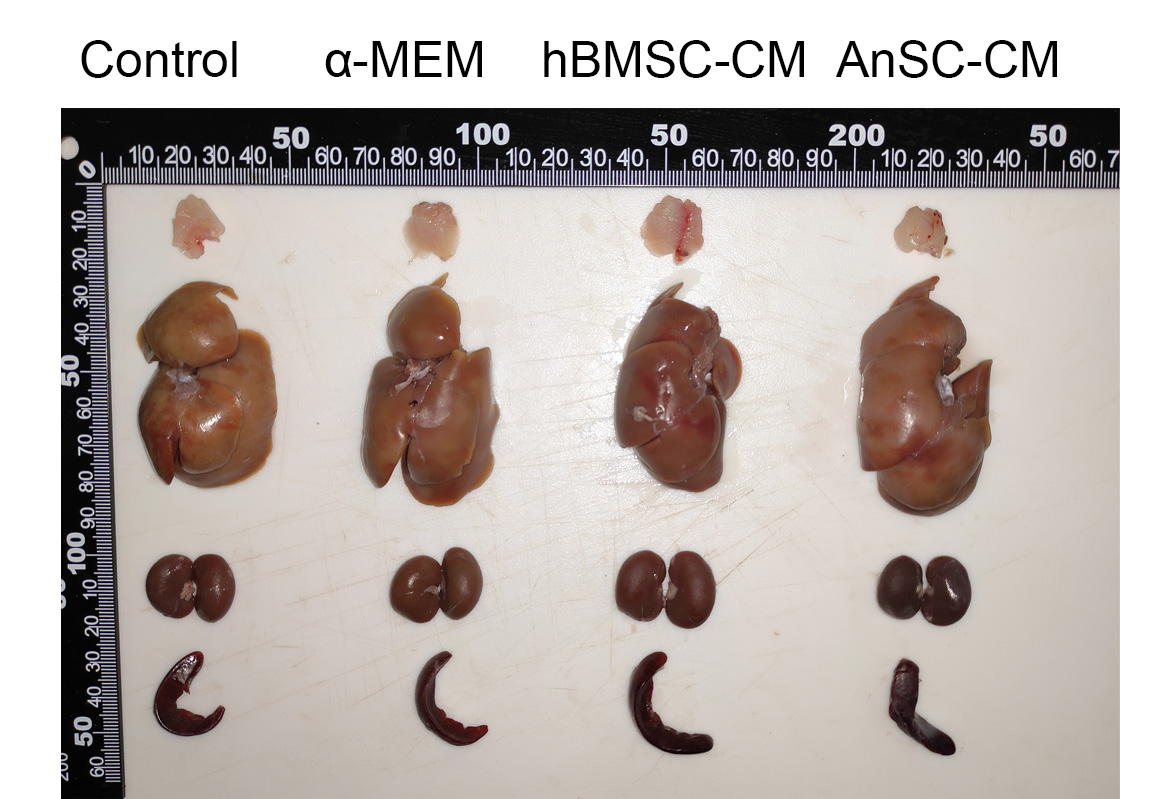

Supplement: Supplementary file 1 — Figure S1. Morphological examination of vital organs including thymus, liver, kidney and spleen. There were no visible abnormities detected in the thymus, liver, spleen and kidney from the AnSC‐CM treated rats. [file CPR-56-e13454-s004.tif]

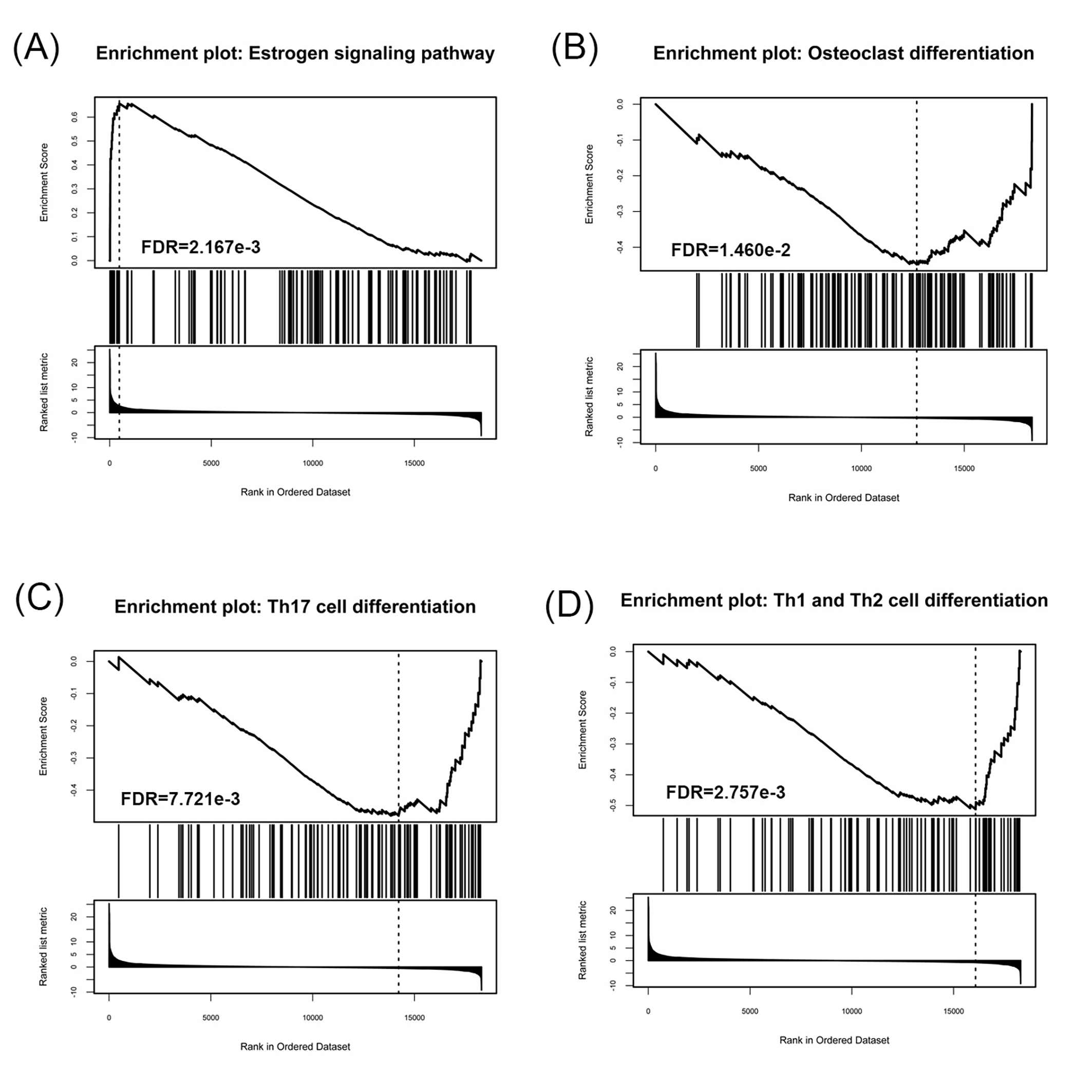

Supplement: Supplementary file 2 — Figure S2. Gene set enrichment analysis (GSEA) of RNA‐seq data. (A) Oestrogen signalling pathway was activated. (B) Osteoclast differentiation, some immune responses were inhibited. (C) Th17 cell differentiation was inhibited. (D) Th1 and Th2 cell differentiation was inhibited. Note that all these were inhibited in the AnSC‐CM group compared with the α‐MEM group. FDR, false discovery rate (adjusted p value). [file CPR-56-e13454-s003.tif]

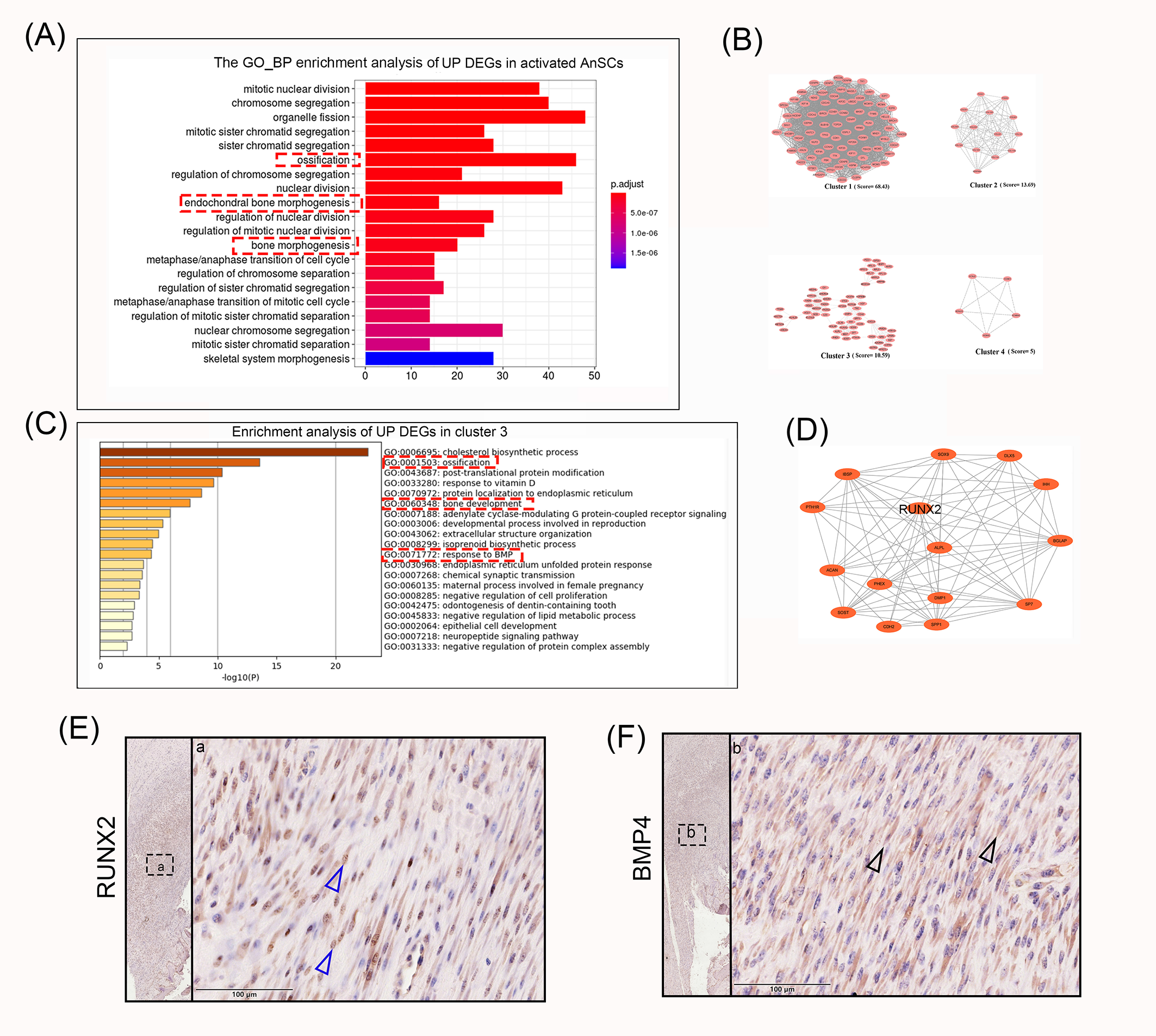

Supplement: Supplementary file 3 — Figure S3. Up‐regulated DEGs in the activated AnSCs are mainly osteogenesis‐related (A) GO analyses of up‐regulated DEGs in the activated antler stem cells (AnSCs). (B) Module analysis of all up‐regulated DEGs. Four clusters were screened with a cut‐off k‐score = 5 depending on the MCODE scoring system. (C) GO analyses of DEGs in the cluster 3. (D) Protein–protein interaction networks for genes in cluster 3. Note that RUNX2 was located at the centre of the network. (E,F) Immunohistochemical staining (IHC) of RUNX2 (E) and BMP4 (F) in the AnSCs. RUNX2 positive staining was mainly localized in the nucleus, while BMP4 positive staining was mainly located in the cytoplasm. Scale bar = 100 μm. [file CPR-56-e13454-s001.tif]
